# Techniques for analysing pattern formation in populations of stem cells and their progeny

**DOI:** 10.1186/1471-2105-12-396

**Published:** 2011-10-12

**Authors:** John A Fozard, Glen R Kirkham, Lee DK Buttery, John R King, Oliver E Jensen, Helen M Byrne

**Affiliations:** 1 Centre for Mathematical Medicine & Biology, School of Mathematical Sciences, University of Nottingham, University Park, Nottingham NG7 2RD, UK; 2 Wolfson Centre for Stem Cells, Tissue Engineering & Modelling, Centre for Biomolecular Sciences, University of Nottingham, University Park, Nottingham NG7 2RD, UK; 3 Division of Advanced Drug Delivery & Tissue Engineering, School of Pharmacy, University of Nottingham, University Park, Nottingham NG7 2RD, UK

## Abstract

**Background:**

To investigate how patterns of cell differentiation are related to underlying intra- and inter-cellular signalling pathways, we use a stochastic individual-based model to simulate pattern formation when stem cells and their progeny are cultured as a monolayer. We assume that the fate of an individual cell is regulated by the signals it receives from neighbouring cells via either diffusive or juxtacrine signalling. We analyse simulated patterns using two different spatial statistical measures that are suited to planar multicellular systems: pair correlation functions (PCFs) and quadrat histograms (QHs).

**Results:**

With a diffusive signalling mechanism, pattern size (revealed by PCFs) is determined by both morphogen decay rate and a sensitivity parameter that determines the degree to which morphogen biases differentiation; high sensitivity and slow decay give rise to large-scale patterns. In contrast, with juxtacrine signalling, high sensitivity produces well-defined patterns over shorter lengthscales. QHs are simpler to compute than PCFs and allow us to distinguish between random differentiation at low sensitivities and patterned states generated at higher sensitivities.

**Conclusions:**

PCFs and QHs together provide an effective means of characterising emergent patterns of differentiation in planar multicellular aggregates.

## Background

**Figure 1:**
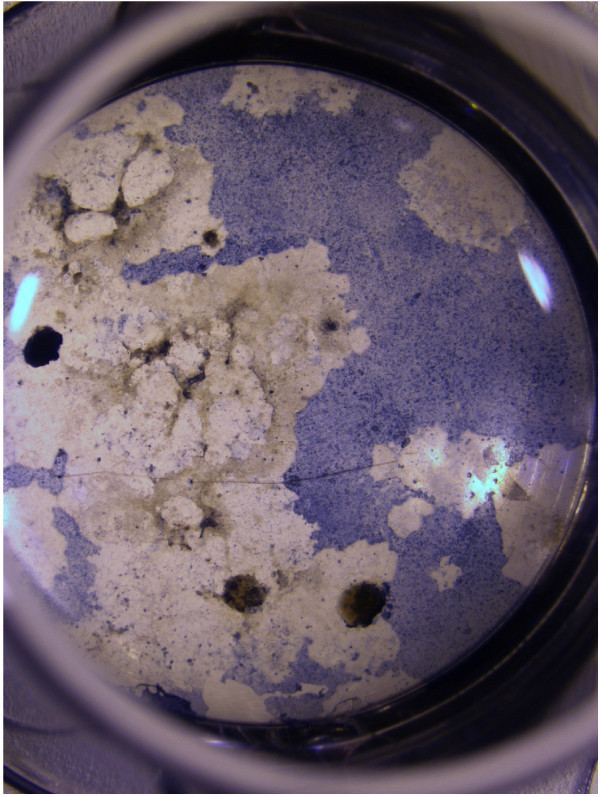
**Patterns of cultured embryonic stem cells**. Photograph showing murine embryonic stem cell aggregates (dark circular objects) adherent to tissue culture plastic and cultured under control conditions. Single cells migrated away from the aggregates, eventually forming a sheet of cells. Alcian blue staining suggests the presence of chondrogenesis.

Embryonic stem cells (ESCs) hold great promise as a source of cells for regenerative medicine, as they are, in principle, capable of being expanded indefinitely *in vitro *and have the potential to differentiate into any adult cell type. Whilst small molecules (such as dexamethasone, vitamin C and retinoic acid [1]), or growth factors (such as bone morphogenesis proteins (BMPs) and transforming growth factor *β *(TGF-*β*) [2]) can be used to increase the proportion of cells of a desired type, the population typically consists of multiple cell types, often organised into distinct patches (as illustrated in Figure 1). Culturing cells for extended periods of time *in vitro *is expensive and stem cells are generally in short supply. There is therefore value in using mechanistic theoretical models of the differentiation of cultured cells to investigate the relationship between the processes determining the fate of individual cells and tissue-scale patterns. Such models can be used to develop optimised protocols for the production of specific cell types and for the development of relevant analytical techniques. In this paper, we present a computational model of a population of stem cells, forming a relatively dense confluent monolayer, in which juxtacrine or diffusive cell signalling biases differentiation of individual cells into two possible cell types. We demonstrate how statistical tools (pair correlation functions and quadrat histograms) can be used to characterise the emergent patterns of differentiation arising from these distinct signalling mechanisms.

In the context of stem-cell differentiation, theoretical models have successfully described for instance the OCT4-SOX2-NANOG system [3], lineage determination between trophectoderm and endoderm [4] and the later differentiation of cells into one of three mesenchymal lineages under the regulation of the master transcription factors RUNX2, SOX9 and PPAR-*γ *[5]. However interactions between multiple pathways remain poorly characterised [5] and many of the key processes involved in cell differentiation remain to be identified. More abstract theoretical models for cellular differentiation are based on the identification of cell fates with distinct attractors of an underlying dynamical system [6]. This idea is embodied in the concept of the 'epigenetic landscape' [7], whereby a ball rolling down a slope into a branching network of valleys is analogous to a differentiating cell choosing between distinct fates. Such ideas have been revisited [8,9] in the light of recent observations of differentiating stem cells. Subsequent work has sought to identify explicitly some of the attractors in the dynamical system generated by the cell's internal regulatory networks [10,11].

The development of mechanistic models to describe pattern formation is a cornerstone of mathematical biology. Substantial attention has focused on systems which exhibit Turing instabilities, involving competition between short-range inhibitors and long-range activators [12]. Such models have been used to describe pattern formation in populations of differentiating cells; for example Garfinkel *et al. *[13] examined the formation of swirls and ridges in populations of mesenchymal cells. A range of alternative mechanisms have also been investigated, in the context of stem-cell differentiation, involving for example the combination of hapotaxis and cell-cell adhesion in mesenchymal condensations leading to the formation of patches of cartilage [14], hapotaxis and activator-inhibitor dynamics combined with a discrete model for cell motion [15] and static activator-inhibitor models [16,17]. As these diverse studies suggest, there are a number of mechanisms by which patches of different cell types could be generated. For example, cells with a similar clonal history are likely to be found near each other, and inherited transcription factors and epigenetic changes may predispose their differentiation into similar types. Alternatively, cells could first differentiate and subsequently organise (or 'sort') themselves into patches through spatial rearrangement [18,19]. The distribution of mechanical forces in the culture environment, or the spatial distribution of chemicals, could favour differentiation into particular cell fates in specific regions of the culture system; and cells may influence the differentiation of their neighbours, by auto/paracrine signalling through diffusive signalling molecules, or by juxtacrine signalling between adjacent cells (possibly mediated by local mechanical effects) [20]. The above list is certainly not exhaustive and it is likely that multiple mechanisms act in combination.

In this paper, we focus on two candidate mechanisms that may be responsible for pattern formation in populations of stem cells and their progeny, considering patterns which are formed by the transmission of information between cells through either diffusible morphogens or juxtacrine signalling, biasing differentiation pathways. Candidate diffusible morphogens might include TGF-*β *and BMP-2, as reviewed in [21], see also [22-24]. We neglect details relating to diffusive transport [25] such as transcytosis [26] or binding of morphogens to cell surfaces or the extracellular matrix. The juxtacrine case could model lateral induction through Notch signalling, which is known to be involved in regulating differentiation and has been found to stimulate the differentiation of embryonic stem cells (ESCs) into neurons [27] and epithelial stem cells into the functioning cells of the intestinal crypt [28]. Alternatively, this case could represent the effects of signalling mediated by cell-cell adhesion molecules such as cadherins [29,30], some of which are thought to modulate differentiation [31].

While our model is generic in the sense that we do not identify explicit morphogens or signalling pathways in our model, we can nevertheless use it to investigate the physical mechanisms that underlie experimentally observed patterns. Previous studies illustrate the complexity of this task. While juxtacrine signalling is typically concerned with pattern formation on the lengthscale of a cell [32], it can exert a longer-range effect. For example, in the imaginal disc of Drosphilia, sensory organ precursor cells extend filopodia containing Delta, allowing them to signal to cells which are not nearest neighbours [33]. Lateral induction of ligand production [34] can generate large-scale patterns, with the juxtacrine signal being relayed between neighbouring cells [35]. Newman & Bhat [36] suggest a mechanism in which oscillatory behaviour synchronised by juxtacrine signalling generated large scale patterns by limiting the period of time over which condensations could grow.

The differences between patterns arising from diffusive and juxtacrine signals therefore merit careful investigation. Given the complexity of modelling specific multi-step differentiation pathways, and their interactions with other signalling networks, we propose here a deliberately simple pattern-generating model that captures generic features in qualitative terms using minimal parameter sets. Motivated by the idea of the epigenetic landscape, we consider a model in which the state of an individual cell evolves as a flow on a two-dimensional surface [11]. The surface branches into two valleys, which correspond to the two alternative cell fates. Differentiating cells are assumed to influence other cells through juxtacrine or diffusive signalling, 'tilting' the potential landscape of a target cell and breaking the symmetry of the pitchfork bifurcation. We assume the bifurcation is supercritical, unlike the subcritical case treated by Huang et al. [37]. We incorporate stochasticity in our model in two ways: by introducing noise into the differentiation process [38,39]; and by introducing a random element to the initial spatial distribution of cells within the monolayer. However, to avoid further complexity, we neglect cell motility and division while differentiation takes place.

In order to analyse the patterns that emerge from our simulations, we employ statistical measures for marked or multitype spatial point processes. One common class of spatial statistics are 'second-order' characteristics, which include Ripley's K-function [40] and pair correlation functions (PCFs) [41], that consider the distribution of distances between pairs of points. Statistics of this class have associated cross or bivariate versions, which only consider distances between pairs of points of specific types. Both the standard and cross-type versions of these statistics have been previously used to examine the distribution of cells in experimental data. For example, a number of statistics, including PCFs, were used by [42] to examine the spatial locations of dividing and non-dividing cells in histological sections of solid tumours. Ripley's K-function [40] has been used to examine retinal neurons [43], the three-dimensional distributions of osteocyte lacunae [44], nerve cells [45], and villous branches in the placenta [46]. Ripley's L-function (a variant of the K-function [40]) was used to examine immune cells in lymph nodes [47]. Su *et al. *[48] use "local cell metrics" (LCMs), which are closely related to PCFs (their normalised LCM is precisely the cross PCF), to analyse cell-cell interactions in populations of proliferating osteoblasts. However, the types of spatial patterns arising in these experiments, and the biological questions under consideration, differ from those considered here. We note that other spatial statistics have been developed, in particular Minkowski functionals [49], which are more complicated to implement than second-order statistics.

In this paper, we examine two statistical measures that are particularly well suited to multicellular systems, and which could equally be applied to experimental observations. These provide a quantitative estimate of pattern length scales in populations of two cell types, distinguish 'noisy patterns' from completely random differentiation and condense image data into a small number of measures which are useful for parameter surveys. We show how PCFs can be used to assign a length-scale to patterns of differentiating cells. We also show how *quadrat histograms *(QH) can be used to distinguish noisy patterns from random distributions. QHs are adopted here on account of their conceptual simplicity, ease of implementation and low computational cost. PCFs were chosen in preference to other second-order statistics because of their (arguably) more natural interpretation in the context of exploratory data analysis, as they indicate the properties of pairs of cells separated by a particular distance (rather than all those pairs separated by less than a given distance, which is the case for the Ripley's K-function). These tools generate simple metrics that enable us to characterise the patterns that emerge and their dependence on system parameters.

## Results

**Figure 2:**
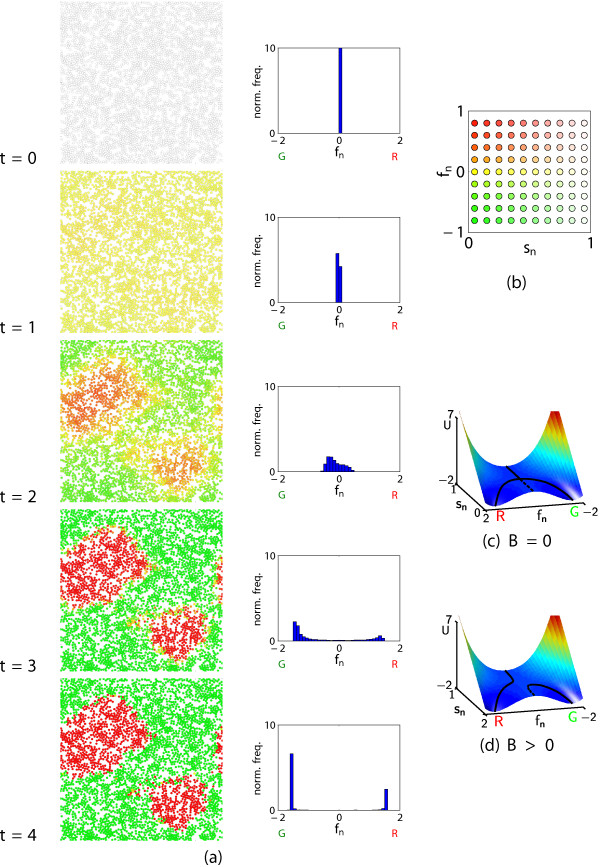
**Temporal development of differentiation patterns**. In (a), the left-hand diagrams show the cell states (*s*_*n*_, *f*_*n*_), with cells coloured according to the key in (b); the right-hand diagrams are the corresponding QHs for the distribution of the cell type variable *f*_*n*_. "norm. freq." is normalized frequency. Parameter values: as in Table 1. (c,d) show the potential surface for (2b), $U=B_{n}s_{n}+\chi \left(\frac{1}{2}-s_{n}\right)f_{n}^{2}∕2-\nu f_{n}^{4}∕4$ for *χ *= 5, *ν *= 1 with (c) *B*_*n *_= 0 and (d) *B*_*n *_= 0.1. The cells start in a multipotent state (upper valley), but as they progress down the surface they diverge into two distinct phenotypes (lower valleys). Diffusive morphogens bias differentiation towards one of the two states ("tilting" the surface). Solid lines correspond to stable steady-states for the type equation (2b) with *s*_*n *_viewed as a constant parameter.

The model is initialised by seeding undifferentiated cells at random on a planar surface, and allowing them to push each other apart at short distances (and attract nearby cells at longer distances) to form aggregates with only minimal overlap between cells. Thereafter (for *t *> 0), the cells are assumed to remain stationary while they undergo differentiation into one of two possible terminal states, denoted R (red) or G (green) (Figure 2(a)). The evolution of each cell is modelled by a stochastic differential equation which is analogous to the motion of a particle (in the presence of noise) down a valley (in a surface with coordinates (*s*_*n*_, *f*_*n*_)) that bifurcates into two sub-valleys via a pitchfork bifurcation (Figure 2(c)). The 'stemness' parameter *s*_*n *_for cell *n *falls from 1 to 0 as the cell differentiates; the type of cell *n *is coded by a variable *f*_*n *_that approaches the base of the sub-valley in *f*_*n *_> 0 (*R*) or *f*_*n *_< 0 (*G*).

Signals from nearby cells tilt the landscape (Figure 2(d)), favouring differentiation towards the fate shared by its neighbours. Noise in the signalling, generated by randomness in the initial spatial distribution of the cell aggregates and intrinsic variation in the differentiation of each cell, leads to the formation of local regions containing more cells of type *R *(*f*_*n *_> 0) or *G *(*f*_*n *_< 0).

Partitioning of the cells into distinct fates is illustrated by histograms of *f*_*n *_(Figure 2(a)). The distributions presented in Figure 2(a) illustrate one of a large set of possible simulation outcomes.

### Characterising patterns

**Figure 3:**
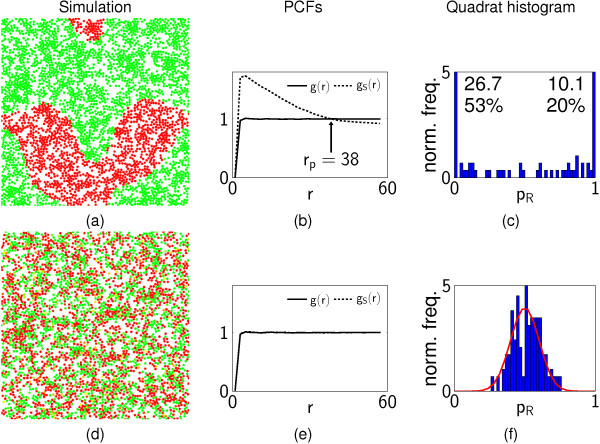
**Characterising patterns with spatial statistics**. (a,d) show two representative model simulations, computed with different parameter values ((a) *S*^diff ^= 40, λ = 10; (b) *S*^diff ^= 1, λ = 40); colours indicate *R/G *differentiation states; non-overlapping disks represent individual cells. (b,e) show the corresponding PCFs and (c,f) the QHs. In (b,e), the dashed line is the cross PCF *g_S_*(*r*) for pairs of cells of the same type; the solid line is the PCF *g*(*r*) for all pairs of cells. The numbers on the QH (*c*) indicate the normalised frequencies (and corresponding percentages) for the end bins (which exceed the vertical scale of the histogram). In (a,b,c), the cells are organised into distinct patches, reflected in the behaviour of the PCFs; *g_S_*(*r*) *> g*(*r*) for *r < r*_*P *_(i.e., nearby pairs of cells are more likely to be of the same type than two cells selected at random), the intersection point *r = r*_*p *_giving a quantitative estimate of pattern scale. The QH (*c*) also indicates the formation of patches, as the majority of the quadrats contain cells of one type (the range 0 <*p *< 1 is divided into 50 bins, and the values are normalised such that the total area is one). In (d,e,f), the cells appear to have differentiated at random, with no discernible structure. This can be seen from the PCFs in (e), with *g_S _*≈ *g*(*r*) for all *r *(i.e., two nearby cells selected at random are no more likely to be of the same type than two well-separated cells). Similarly, the QH (f) shows that most of the quadrats contain a mixture of cells of different types, and the proportion of cells of type *R *in each quadrat is well described by a truncated normal distribution on [0,1] with mean 1/2 and variance $M_{q}^{2}∕4N$ (solid line).

At the end of each simulation, cells are characterised by the positions of their centre and their type (*R *or *G*). Two representative patterns are shown in Figure 3, with which we illustrate the use of PCFs and QHs.

PCFs are represented by two functions, *g*(*r*) and *g_S_*(*r*): *g*(*r*) describes the distribution of distances *r *between pairs of cells, normalised by the expected distribution if the cell positions were completely random; the cross-PCF *g_S_*(*r*) represents the distribution of distances between pairs of cells of the same type (either *R *or *G*). If the cells have a completely random spatial distribution, then *g*(*r*) ≡ 1 (although the requirement for cells not to be overlapping implies *g *< 1 for small *r*). If cells differentiate randomly and independently, then *g_S_*(*r*) ≡ *g*(*r*) (Figure 3(e)). However, if the cells form patches of different types, then the cross PCF will differ from *g*(*r*) (Figure 3(b)). For example, for distances *r *smaller than the sizes of the patches, *g_S_*(*r*) >*g*(*r*), as two cells separated by a distance r are more likely to be of the same type than two cells which are selected at random. The point at which the PCFs intersect (*r *= *r*_*p *_≈ 38 in this case) provides a quantitative estimate of the scale of the pattern.

QHs indicate the proportion *p*_*R *_of cells of type *R *in each quadrat when the domain is divided into *M*_*q *_× *M*_*q *_square quadrats. If the cells differentiate at random (Figure 3(d,e,f)), and the number of quadrats is chosen such that the average number of cells in a quadrat $N∕M_{q}^{2}$ is moderately large ($N∕M_{q}^{2}>10$), then *p*_*R *_has an approximately binomial form, *N*_*q*_*p_R _*~ *B*(*N*_*q*_, 1/2) with $N_{q}=\left\lfloor N∕M_{q}^{2}\right\rfloor$; there are on average *N*_*q *_cells in each quadrat, and the type of each cell is determined randomly and independently of the others with probability $\frac{1}{2}$ of being *R*. For large *N, p*_*R *_is approximately normally distributed with $p_{R}\sim N\left(1∕2,1∕4N_{q}\right)$ (Figure 3(f)). However, if there are distinct regions (with a length scale larger than the size of the quadrats) in which most cells are of one type then there will be many quadrats for which *p *≈ 0 and *p *≈ 1, resulting in a distribution with two large peaks (Figure 3(c)). Thus distributions with distinct patches are identified by PCFs with *g_S_*(*r*) >*g*(*r*) for sufficiently small *r *and QHs showing a substantial majority of quadrats containing cells which are almost all of one type. In contrast, spatially random patterns of differentiation (as illustrated in Figure 3(d,e,f)) are characterised by *g_S_*(*r*) ≈ *g*(*r*) and a QH of binomial form.

In summary, QHs provide simple information about whether or not a pattern is present whereas PCFs provide additional information about the pattern's length-scale.

### Diffusive signalling

**Figure 4:**
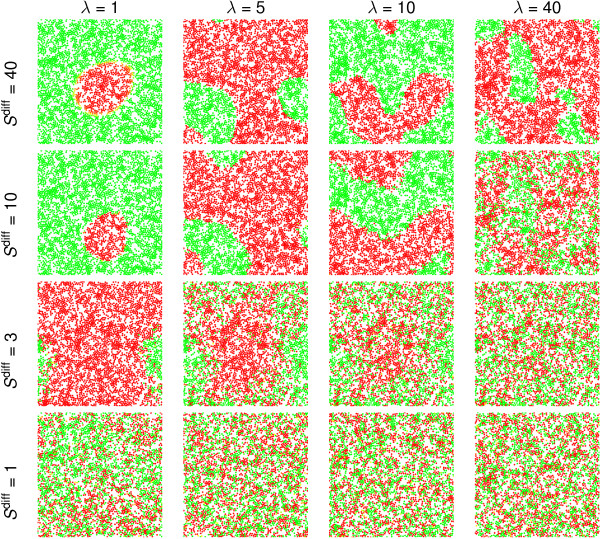
**Survey of patterns under diffusive signalling**. Patterns generated by the diffusive signalling mechanism, for a variety of different values of the sensitivity parameter, *S*^diff ^= 1,3,10,40 and the morphogen decay rate, λ = 1,5,10,40, as indicated. We plot the distribution of cells at the end of a single realisation of the model for each pair of values *S*^diff ^and λ. For fixed *S*^diff^, increasing λ decreases the scale of the patterns. For fixed λ, as *S*^diff ^increases there is a transition from random differentiation, through a "noisy pattern" stage, to patches of cells which are almost all of one type. The point at which this transition occurs depends upon the values of both parameters.

The spatial patterns that are observed under diffusive signalling are particularly sensitive to two dimensionless model parameters: *S*^diff^, which measures the response of the bias to morphogen concentrations; and the morphogen decay rate, λ. Results from individual realisations of the model for 16 pairs of parameter values are shown in Figure 4, illustrating the range of patterns that can be generated. For small *S*^diff ^and large λ, the cells appear to differentiate randomly, as the strong decay rate inhibits communication between cells. For large *S*^diff ^and small λ, the patterns often contain many more of one cell type than another, and in some cases all cells adopt the same (differentiated) fate, with stochastic effects dictating whether they are all red (of type *R*) or all green (of type *G*). For fixed λ and increasing *S*^diff^, we observe a transition from random differentiation to distinct patches of cells, with "noisy patches" evident for intermediate values of *S*^diff^; patterning is more coherent when cells have greater sensitivity to morphogens. For fixed *S*^diff ^and increasing λ, the spatial scale of the patches appears to decrease, with the differentiation becoming random for sufficiently large λ.

**Figure 5:**
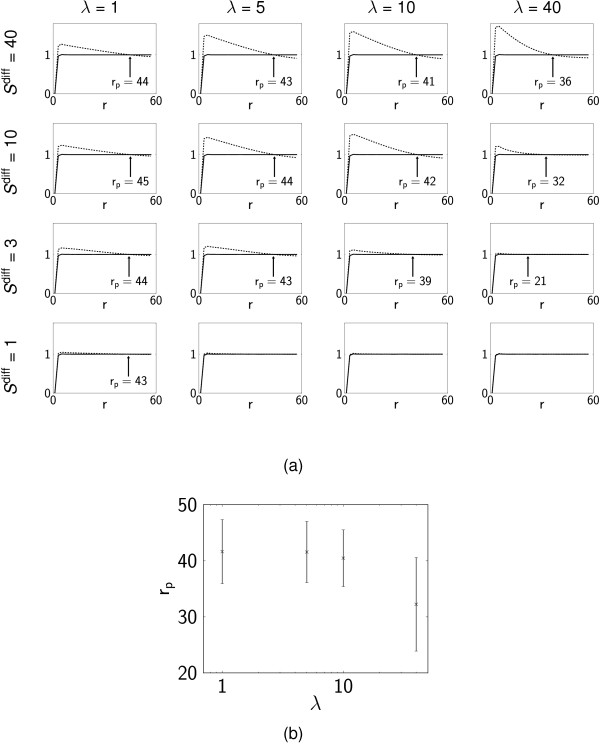
**Pair correlation functions: diffusive signalling**. (a) PCFs for simulations with diffusive signalling. For each set of parameter values PCFs are calculated using the results of *M*_sim _= 100 realisations (corresponding to the individual realisations shown in Figure 4). The dashed line is the cross PCF *g_S_*(*r*) for pairs of cells of the same type whilst the solid line is the PCF *g*(*r*) for all pairs of cells. Arrows highlight *r*_*p*_, the point of intersection of *g_S _*and *g*, which is a quantitative estimate of pattern size. (b) Distribution of the values of *rp *calculated from each individual realisation of the simulation with *S*^diff ^= 10 and λ = 1,5,10,40 (error bars show mean plus or minus one standard deviation). Only realisations in which the maximum value of *g_S _- g *is greater than 0.02 (ignoring those realisations in which all cells differentiate to the same type) are included; for λ = 1, 5,10,40 this corresponds to *n *= 43, 78, 96,100 of 100 realisations, respectively.

To identify behaviour that is consistent across multiple realisations, simulations were conducted *M*_sim _= 100 times for each parameter set in Figure 4. The corresponding PCFs, averaged over all simulations (Figure 5(a)), demonstrate consistently random differentiation for small values of *S*^diff ^and large λ (*g_S_*(*r*) ~ *g*(*r*)). Distinct patches are evident for larger *S*^diff ^and small λ (*g_S_*(*r*) >*g*(*r*) for *r *<*r*_*p*_). The quantitative estimates of the scale of the pattern, *r*_*p*_, increase slightly as λ decreases (the diffusive signals act over distances proportional to $\sqrt{D∕\lambda }$), Figure 5(b), but are less sensitive to *S*^diff^. We report values of *r*_*p *_for the mean PCFs in Figure 5(a), noting that there is a distribution of patch sizes between individual simulation realisations; the width of this distribution is indicated in Figure 5(b). The difference between *g_S_*(*r*) and *g*(*r*) becomes smaller for small λ, because some realisations contain cells which are all of one type (in which case *g_S_*(*r*) ≡ *g*(*r*)).

**Figure 6:**
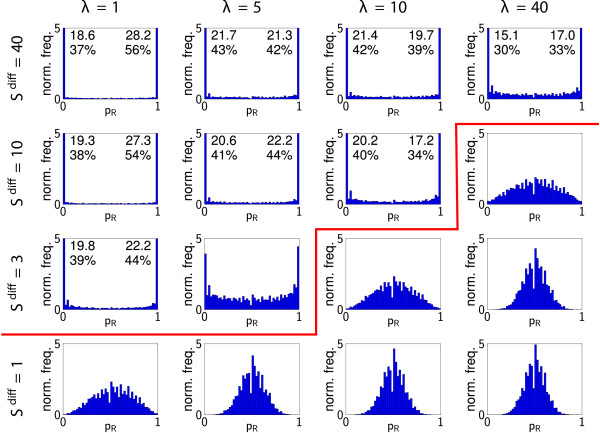
**Quadrat histograms: diffusive signalling**. QHs for simulations with diffusive signalling. For each set of parameter values histograms are calculated using the results of *M*_sim _= 100 realisations (corresponding to individual realisations shown in Figure 4). Numbers show normalised frequencies (and corresponding percentages) for the bins if these are greater than 5. In those QH to the upper-left side of the red line, more than 10% of the quadrats are in either of the extreme bins (*p*_*R *_< 0.02, *p*_*R *_> 0.98), which we use as a conservative criterion for the presence of patterns.

The corresponding QHs (averaged over *M*_sim _realisations, see Figure 6), demonstrate a transition from random differentiation for small *S*^diff ^and large λ, in which the histogram has a binomial form with a peak at *p = *1/2, to well-defined patterns for large *S*^diff ^and small λ in which the majority of the quadrats contain cells which are entirely of one type (*p*_*R *_≈ 0,1). It is helpful to introduce a (very conservative) threshold that defines the existence of patterns: for example, if more than 10% of the quadrats have *p*_*R *_< 0.02 or *p*_*R *_> 0.98 (so lie in either of the extreme bins of the QH), then we say that well defined patterns exist. We demarcate patterned and non-patterned distributions defined by this criterion in Figure 6. Note that the presence of any quadrats with extreme values of *p *strongly suggests the presence of patterning: with the parameters of Table 1, the average quadrat contains about 24 cells, and if these all differentiate randomly and independently the probability of all 24 being of one type is roughly 2 × (0.5)^24 ^≈ 10^-7^. The degree of noise in the patterns is characterised by the shape of the histograms for intermediate values of *p*_*R*_; the roughly uniform distribution on 0 <*p*_*R *_< 1 falls in magnitude as *S*^diff ^increases (Figure 6), even though pattern length-scales remain approximately constant relative to the size of quadrats (Figure 5). This diffusive signalling mechanism is therefore capable of generating a wide range of spatial patterns. Overall, the sensitivity parameter, *S*^diff^, appears to control the degree of noise in the patterns, whilst the morphogen decay rate, λ, controls their length-scale.

**Table 1:** Dimensionless parameter estimates

Symbol	Description	Dimensionless value
***L***	Computational domain size	120
*N*_init_	Initial number of cells	3500
*r*_*c*_	Typical cell radius	1
*t*_end_	Duration of the simulation	4

*χ*	Bifurcation control parameter	5
*δ*	Noise amplitude	10^-4^

*S*^juxt^	Sensitivity parameter (juxtacrine)	10^-3^
*R*^juxt^	Radius for juxtacrine signalling	3

*S*^diff^	Sensitivity parameter (diffusive)	10
*λ*_a_, *λ*_*b*_	Morphogen degradation rates	10
*D*_*a*_, *D*_*b*_	Morphogen diffusion coefficients	10^3^
*M*_*s*_	Number of grid squares	120

*M*_*q*_	Number of quadrats in each direction	12
*M*_sim_	Number of simulation realisations	100
*M*_*g*_	Number of distance intervals for RDFs	60
d*t*	Time step	4 × 10^-4^

Dimensionless parameter estimates; unless otherwise stated (in the figure caption), these are the parameter values used for simulations.

### Juxtacrine signalling

**Figure 7:**
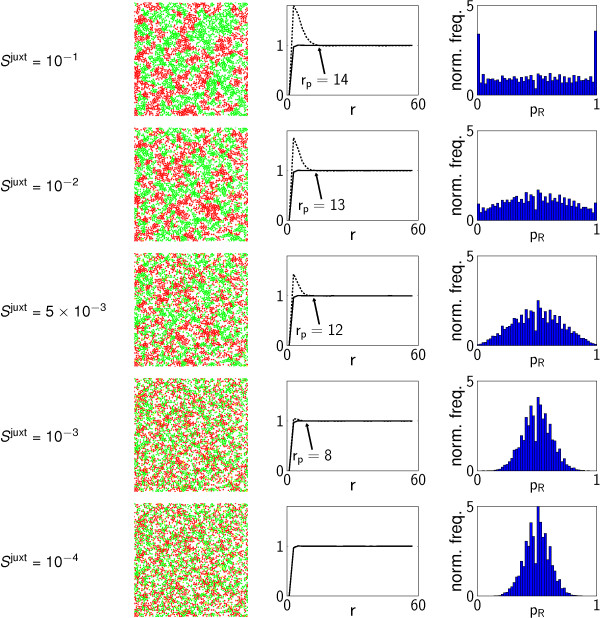
**Juxtacrine signalling patterns**. Patterns generated by the juxtacrine signalling mechanism, for a range of values of the sensitivity parameter, S^juxt^. Each of the quadrat histograms and PCFs was calculated from the results of *M*_sim _= 100 realisations with the same parameter values.

For the juxtacrine signalling mechanism, we consider only the effects of varying the sensitivity parameter, *S*^juxt^. Simulation results (Figure 7) show a smooth transition from random differentiation for small *S*^juxt ^to small, distinct patches of cells for larger *S*^juxt^. In contrast to the diffusive signalling mechanism, patch size under juxtacrine signalling is limited to approximately 20 cell radii in scale. The transition from random differentiation is evident in PCFs (*g_S_*(*r*) ≈ *g*(*r*) for small *S*^juxt^; *g_S_*(*r*) >*g*(*r*) for *r < r*_*p *_for larger *S*^juxt^), which indicate a patch size of approximately *r_p _*≃ 14 for large *S*^juxt^. The QHs also reflect this transition, although as the scale of the patterns is comparable to that of the quadrats, there are substantially fewer quadrats containing cells entirely of one type (*p*_*R *_≈ 0,1) than in the diffusive case (with large *S*^diff ^and small λ).

## Discussion

Heterogeneity in differentiating populations of stem cells hinders the efficient generation of specific types of differentiated cells. Whilst it seems likely that cells will always need to be sorted before being implanted *in vivo*, not least because undifferentiated cells can cause teratomas (e.g. [50]), improving the yield of particular cell lineages would be of great value. The detailed mechanisms which govern the later stages of cell differentiation into particular phenotypes are not well understood, and there is evidence to suggest that components of both diffusible and juxtacrine signalling pathways play a role [21-24,27,28].

The statistical measures described here provide a robust, quantitative measure of noisy spatial patterns. We have shown, using a simple model of diffusive or juxtacrine signalling in a cellular monolayer, how QHs provide a simple measure for distinguishing binary patterns of cellular differentiation from spatially uncorrelated outcomes, and how PCFs may be used to estimate the typical lengthscale of binary patterns. As discussed below, these could be readily applied to experimental data, allowing the objective comparison of patterns associated with different culture conditions. In the future, such measures may prove useful in future for comparing the outputs of mechanistic, theoretical models with experimental outcomes. Spatial multicellular simulations often contain large numbers of parameters and generate verbose output; PCFs and QHs may prove to be useful tools for the automatic exploration of parameter space and for condensing the information into a smaller number of physically meaningful quantities.

### Model extensions

The present model is deliberately simple, but sufficient to capture the fundamental dynamics (a pitchfork bifurcation with symmetry broken by signalling) that we expect to govern cell fate specification. There are many ways in which we could extend the model. For example, we could include more detailed models of the regulatory networks that govern differentiation [5], and details of their interactions with signalling pathways, such as Wnt signalling [51,52], which is thought to play a role in regulating mesenchymal differentiation [53] and the cell fate of intestinal epithelial cells [54].

At present, all cells lose their "stemness" at the same, pre-determined rate. It seems plausible that individual cells could undergo a rapid, asynchronous transition from an undifferentiated stem-like state to a committed or differentiated one; our model could be extended to permit this by changing the form of the potential surface. This would also permit small numbers of partially-differentiated cells to be present in the terminal population [55].

In addition, embryonic stem cell populations have been found to be heterogeneous, containing subpopulations which are biased towards particular lineages [56-58]. Such effects could be modelled by considering a subcritical pitchfork bifurcation, as in the model of [37], rather than the supercritical one considered here. While the current model allows limited plasticity in cell fate, with partially differentiated cells being able to change cell type, it is possible to include de-differentiation in response to specific extracellular signals [59,60] and transdifferentiation of cells [61,62].

More accurate models for diffusive signalling could be developed that account for realistic cell shapes in three dimensions and the details of receptor-ligand binding [63] and signal transduction [64]. The model for juxtacrine signalling could also be greatly refined, incorporating established mechanisms [65-68]. Mechanical forces are also known to affect tissue morphogenesis (reviewed by [69]); changes in cell shape [70] and substrate stiffness [71] have been found to cause mesenchymal stem cells to commit to different lineages. Extracellular matrix (ECM) proteins are thought to regulate differentiation [72-75], and it has recently been observed that the ECM generated by osteogenic precursors promotes the osteogenic differentiation of ESCs [76]. Such effects could be incorporated in a similar manner to diffusible morphogens, but without diffusion. Other extracellular stimuli that are known to influence differentiation, such as O_2 _tension [77,78], could also be readily incorporated in the model.

Cell motion can be readily included in the model, e.g. equation (1), which is here used to determine initial cell positions, could be employed and noise added to account for random cell motility. It would also be interesting to extend the model to account for cell division. However, we have concentrated on the case of static populations of non proliferating cells in order to investigate the two patterning mechanisms in a simple context.

### Applications to experimental data

The positions of the cell nuclei (possibly obtained through DAPI staining and confocal imaging, followed by image segmentation and identification of the centroids of the nuclei) give a set of points in space, and if a cell type can be assigned to each point (through co-staining), the data will be of the same form as that analysed in this paper. The PCFs (and also the QHs) may be calculated in a straightforward manner using the *R *package spatstat [79,80].

## Conclusions

We have shown how two statistical techniques, QHs and PCFs, can be used to analyse the spatial patterns that emerge in populations of differentiating cells, when there is randomness in the spatial distribution of cells and in the superimposed patterns of differentiation. We have illustrated these techniques using data from a simple stochastic model, in which cell patterning is regulated by either diffusive or juxtacrine signals. We have shown how the size and onset of patterns can be quantified, and illustrated how patterns depend on the mechanisms controlling differentiation and the system parameters.

Our results suggest that when diffusive signalling regulates differentiation, pattern size, as characterised by the QHs and PCFs, is strongly influenced by morphogen decay rate and the degree to which the morphogen biases cell differentiation, with large-scale patterns observed when the decay rate is low and the cells' sensitivity to the morphogen is high. For juxtacrine signalling, the size of the patterns that emerge is an increasing, saturating function of the cells' sensitivity to signalling; large-scale juxtacrine patterns were not seen in our simulations. Our results also reveal how standard statistical techniques such as PCFs and the QH may be used to analyse and characterise the patterns that emerge from differentiating populations of cells in planar multicellular aggregates.

## Methods

**Figure 8:**
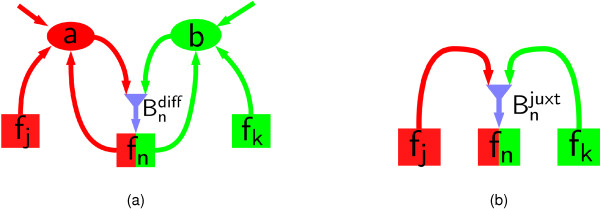
**Pathways of diffusive and juxtacrine signalling**. (a) In diffusive signalling, cells of type *R *(*G*) generate morphogens *a *(*b*) which diffuse in space and influence other cells through a signal $B_{n}^{\text{diff}}$ which biases subsequent differentiation. (b) In juxtacrine signalling, cells of type *R *(*G*) force a signal $B_{n}^{\text{just}}$ which acts on neighbouring cells.

We simulate individual cells on a planar substrate. The model operates in two steps, described in detail below: undifferentiated cells are seeded at random (at *t *= 0), and a mechanical model is used which generates aggregates of non-overlapping cells (at *t *= 0); thereafter (for *t *> 0), individual cells stop moving and undergo differentiation, mediated by diffusive or juxtacrine signalling (see Figure 8). We combine an individual-based model for cell differentiation with a model for signalling; for diffusive signalling, we use continuum reaction-diffusion equations for the diffusible species, whilst for juxtacrine signalling, we assume that each cell influences the differentiation of a finite number of nearby cells.

Patterns of aggregation and differentiation are analysed with PCFs and QHs, as explained below.

### Modelling initial spatial distribution

*N *cells are distributed randomly on a square domain [0, *L*] × [0, *L*], considered to be periodic in both directions. Cells move according to a simple, cell-centre based model for a time interval *t*_init_, generating a distribution that minimises overlapping but allows aggregate formation. Cells move due to forces between neighbouring cells that are repulsive over short distances to prevent overcrowding but attractive over longer distances to mimic adhesion.

The location of the centre of the *n*-th cell, **x**_*n*_, evolves according to the differential equation

(1a) $$\frac{\text{d}x_{n}}{\text{d}t}=\overset{N}{\underset{m=1,m\neq n}{\sum }}v\left(\left|x_{n}-x_{m}\right|\right)\frac{x_{n}-x_{m}}{\left|x_{n}-x_{m}\right|}\cdot$$

Short-range repulsion and long-range attraction are simulated by the velocity *v*(*r*), satisfying

(1b) $$v\left(r\right)=\left\{\begin{array}{cc} Ar_{c}^{2}r^{-3}\left(2r_{c}-r\right) & r<R_{v},\\ 0 & r>R_{v}. \end{array}\right)$$

(We note that other functions having a similar quantitative form would be similarly effective.) We take the cut-off radius to be *R*_*v *_= 3*r*_*c*_, where *r*_*c *_is the cell radius. *A *parametrises the size of cell-cell forces. Equations (1) were simulated using the Euler method for an interval *t*_init _= 0.002, taking *A = *5000.

### Modelling cell differentiation

We parametrise the state of the *n*-th cell (1 ≤ *n *≤ *N*) by (*s*_*n*_, *f*_*n*_), which serves as a low-dimensional approximation to the levels of numerous transcription factors and the methylation status of many genes. The variable *s*_*n*_, lying in the range 0 ≤ *s*_*n *_≤ 1, denotes the "stemness" or degree of plasticity of the cell; each value of *s*_*n *_may represent a set of regulatory network activation patterns from the molecular viewpoint, and may depend on the relative abundance and subcellular localisations of proteins and RNAs as well as other types of signalling molecules.

At the start of the simulations, all cells have stemness parameter *s*_*n *_= 1. Over time and as the cells differentiate, *s*_*n *_decreases (in the present model in a deterministic manner). The variable *f*_*n *_(a measure of the relative expression level of specific genes) may take any real value and represents the differentiation fate of the cells. We classify the cells into two types, *R *and *G*, for which *f*_*n *_> 0 and *f*_*n *_< 0, respectively. (In images of simulations, cells of types *R *and *G *are coloured red and green, respectively.) At the start of the simulation, we set *f*_*n *_= 0 (no preferred lineage) for all cells.

The state of the *n*-th cell evolves according to the system of stochastic ordinary differential equations

(2a) $$\text{d}s_{n}=-\kappa s_{n}\text{d}t$$

(2b) $$\text{d}f_{n}=\left(B_{n}+\chi \left(\frac{1}{2}-s_{n}\right)f_{n}-vf_{n}^{3}\right)\text{d}t+\sqrt{2\delta }\text{d}W_{n}$$

where *t *is time, *κ *> 0 controls the rate at which cells differentiate, while *χ *> 0 and *ν *> 0 are parameters which regulate positive and negative feedback. The equation for *f*_*n *_is chosen such that (with *s*_*n *_viewed as a parameter, and *B*_*n *_= *δ *= 0) it displays a supercritical pitchfork bifurcation at *s*_*n *_= 1/2, with a single stable steady state for *s*_*n *_> 1/2, but two stable (and one unstable) steady states for *s*_*n *_< 1/2, associated with the two distinct cell fates (Figure 2(c)). $B_{n}\equiv B_{n}^{\text{juxt}}+B_{n}^{\text{diff}}$ denotes the influence of external factors (juxtacrine and diffusive signalling) on the fate of the cell. Non-zero *B*_*n *_breaks the symmetry of the pitchfork bifurcation (Figure 2(d)). Noise (of amplitude *δ*) accounts for randomness in the differentiation process, allows plasticity in the fate of partially committed cells, and perturbs the system from the unstable state in which all cells have *f*_*n *_= 0. Cells are assumed to remain stationary while they differentiate. We do not claim that the present model for differentiation is definitive; however, it exemplifies in a simple phenomenological way the phenotypic evolution of individual cells.

#### Diffusive signalling

To simulate diffusive signalling, we assume that the cells produce morphogens with concentrations (at a point **x **in space) denoted by *a*(**x**, *t*) and *b*(**x**, *t*). Cells of type *R *(*f*_*n *_> 0) produce *a*, whilst cells of type *G *(*f_n _*< 0) produce *b*, with the production rates of the *n*th cell being given by *α*_*a*_(*s*_*n*_, *f*_*n*_) and *α_b_*(*s*_*n*_, *f*_*n*_), respectively (Figure 8(a)). The morphogens diffuse freely in the extracellular space, with diffusion coefficients *D*_*a *_and *D*_*b*_, and are degraded at rates λ_*a *_and λ_*b*_. The concentrations *a *and *b *satisfy the equations

(3a) $$\frac{\partial a}{\partial t}=D_{a}\nabla^{2}a+\overset{N}{\underset{n=1}{\sum }}\alpha_{a}\left(s_{n},f_{n}\right)\delta \left(x-x_{n}\right)-\lambda_{a}a$$

(3b) $$\frac{\partial b}{\partial t}=D_{b}\nabla^{2}b+\overset{N}{\underset{n=1}{\sum }}\alpha_{b}\left(s_{n},f_{n}\right)\delta \left(x-x_{n}\right)-\lambda_{b}b$$

where the **x**_*n *_(*n *= 1,..., *N*) are the positions of the cell centres. Uptake of the morphogens by the cells is neglected. For simplicity we adopt the following forms for the production functions:

(3c) $$\alpha_{a}\left(S_{n},f_{n}\right)=\left\{\begin{array}{cc} \alpha \left(1-s_{n}\right) & f_{n}>0,\\ 0 & f_{n}<0, \end{array}\right)$$

(3d) $$\alpha_{b}\left(s_{n},f_{n}\right)=\left\{\begin{array}{cc} 0 & f_{n}>0,\\ \alpha \left(1-s_{n}\right) & f_{n}<0, \end{array}\right)$$

where *α *> 0 is a constant. Production rates increase as the cells lose their multipotency (i.e. as *s*_*n *_decreases).

The influence of morphogens on cell fate in (2b) is modelled by assuming that $B_{n}^{\text{diff}}$ is proportional to the difference in concentrations of the two morphogens,

(3e) $$B_{n}^{\text{diff}}=S^{\text{diff}}\left(a\left(x_{n}\right)-b\left(x_{n}\right)\right),$$

*S*^diff ^being a parameter representing the sensitivity of cells to diffusive signalling. Differentiation is biased towards type *R *(*G*) when $B_{n}^{\text{diff}}$ is positive (negative) via (2b).

#### Juxtacrine signalling

To simulate signalling between cells which are in direct physical contact (represented by cells whose centres are less than a distance *R*_juxt _apart, where we take *R*_juxt _= 3*r*_*c*_), we define the influence function $B_{n}^{\text{juxt}}$ in (2b) to be

(4a) $$B_{n}^{\text{juxt}}=S^{\text{juxt}}\underset{m}{\sum }\frac{2r_{c}\left(\beta_{a}\left(s_{m},f_{m}\right)-\beta_{b}\left(s_{m},f_{m}\right)\right)}{\left|x_{m}-x_{n}\right|}$$

summing over all *m *≠ *n*, with | **x**_*m *_- **x**_*n *_|<*R*_juxt_. The signals produced by differentiating cells (Figure 8(b)) are chosen to be

(4b) $$\beta_{a}\left(s_{n},f_{n}\right)=\left\{\begin{array}{cc} \beta \left(1-s_{n}\right) & f_{n}>0,\\ 0 & f_{n}<0, \end{array}\right)$$

(4c) $$\beta_{b}\left(s_{n},f_{n}\right)=\left\{\begin{array}{cc} 0 & f_{n}>0,\\ \beta \left(1-sn\right) & f_{n}<0. \end{array}\right)$$

*S*^juxt ^parametrises the sensitivity of cells to juxtacrine signalling and the constant *β *> 0 represents the typical number of cell-surface ligands. In (4a), the area of contact between cells (and hence the number of receptor-ligand interactions) is assumed to be inversely proportional to the distance between them.

#### Parameter estimation and nondimensionalization

The governing equations can be simplified by making the model dimensionless. The parameters *r*_*c*_, *κ*, *α*, *β *and *ν*, can be eliminated by rescaling time on *κ*^-1^, distances on *r*_*c*_, the cell fate variable *f*_*n *_on *κ*^1/2^*ν*^-1/2^, diffusive morphogen concentrations and production rates on $\alpha ∕\kappa r_{c}^{2}$ and *α *respectively, juxtacrine production rates on *β *and biasing functions *B*_*n *_on *κ*^3/2^*ν*^-1/2^. In dimensionless variables, we recover equations (2) with *κ *= *ν *= 1 and parameters *χ *and *δ *replaced by $\hat{\chi }=\chi ∕\kappa$ and $\hat{\delta }=\delta \nu ∕\kappa^{2}$; equations (3) with *D*_*a*_, *D*_*b *_replaced by ${\hat{D}}_{a}=D_{a}∕\kappa r_{c}^{2}$, ${\hat{D}}_{b}=D_{b}∕\kappa r_{c}^{2}$ and λ_*a*_, λ_*b *_replaced by ${\hat{\lambda }}_{a}=\lambda_{a}∕\kappa$, ${\hat{\lambda }}_{b}=\lambda_{b}∕\kappa$; equations (3d) with *α *= 1; equation (3e) with *S*^diff ^replaced by ${Ŝ}^{\text{diff}}=S^{\text{diff}}\alpha \nu^{1∕2}∕\kappa^{5∕2}r_{c}^{2}$; equation (4a) with *r*_*c *_= 1 and *S*^juxt ^replaced by ${Ŝ}^{\text{juxt}}=S^{\text{juxt}}\beta \nu^{1∕2}∕\kappa^{3∕2}$ and *R*_juxt _by $R_{\text{juxt}}=R_{\text{juxt}}∕r_{c}$; and equations (4b,c) with *β *= 1. The domain becomes $\left[0,\hat{L}\right]\times \left[0,\hat{L}\right]$ with $\hat{L}=L∕r_{c}$, and simulations are of duration ${\hat{t}}_{\text{end}}=\kappa t_{\text{end}}$. Henceforth we work only with dimensionless quantities and omit hats.

Estimates for the dimensionless parameters are listed in Table 1; these are the default values used for simulations in Results. *D*_*a *_and *D*_*b *_are based on the diffusion coefficient for the morphogen BMP-2, which was estimated to be 10^-8 ^cm^2^s^-1 ^in [13] (we do not include the correction proposed in [13] for the slowing of diffusion by the extracellular matrix), and we take *D*_*a *_*= D*_*b*_. The typical cell radius is taken to be 10 *μ*m. Data to estimate the other parameters are not readily available, in particular *κ*, which we take to be *κ *= 1 day^-1^. However the parameters *S*^diff^*, S*^juxt ^and λ_*a*_, λ_*b *_have a significant effect on the generated patterns, and therefore a wide region of parameter space is surveyed. (We note that the range of λ considered (1 ≤ λ ≤ 40) encompasses the degradation rate 2.5 × 10^-4 ^s ^-1 ^for the morphogen Dpp in Drosophila measured by [81], corresponding to λ = 21 in dimensionless units.) For simplicity we assume λ_*a *_= λ_*b *_= λ, say.

In order to select parameter values such that the diffusive and juxtacrine mechanisms exert similar effects on differentiating cells, we estimate the maximum sizes of $B_{n}^{\text{diff}}$ and $B_{n}^{\text{just}}$. Cells are typically separated from their nearest neighbours by a dimensionless distance of 2 (2*r*_*c *_in dimensional units), so for the juxtacrine mechanism the contribution to $B_{n}^{\text{just}}$ in (4) from a neighbouring cell is of the order of *S*^juxt^. As cells typically have 6 or fewer neighbours (close packing for discs), we estimate $|B_{n}^{\text{juxt}}|\approx 6S^{\text{juxt}}$. For the diffusive signalling mechanism, the steady-state morphogen field generated by a point source of strength unity is given by

(5) $$a=\frac{1}{2\pi D_{a}}K_{0}\left(\sqrt{\frac{\lambda_{a}}{D_{a}}r}\right)$$

where *r *is the distance from the source and *K*_0 _a modified Bessel function. As $K_{0}\left(x\right)\sim e^{-x}\sqrt{\pi ∕2x}$ as *x *→ ∞, diffusive signalling will be significant between cells separated by $r=O\left(\sqrt{D_{a}∕\lambda_{a}}\right)$. Provided λ_*a *_≪ *D*_*a*_, we estimate

(6) $$|B_{n}^{\text{diff}}|\approx \frac{S^{\text{diff}}\phi }{D_{a}}\int_{0}^{\infty }K_{0}\left(\sqrt{\frac{\lambda_{a}}{D_{a}}}r\right)r\;dr=\frac{S^{\text{diff}}\phi }{\lambda_{a}}$$

where $\phi =1∕\left(2\sqrt{3}\right)$ represents the density of cell centres for closely packed discs. For *D*_*a *_= 1000, λ_*a *_= 10, this expression is approximately 0.03*S*^diff^. We therefore expect that the juxtacrine and diffusive signalling mechanisms will have similar effects on differentiation if *S*^juxt ^is roughly 1000 times smaller than *S*^diff^.

#### Numerical methods

Solutions to the stochastic differential equations (2) are approximated numerically using the Euler-Maruyama method [82]. Denoting by Δ*t *the integration timestep and introducing the superscript *τ *to represent the state of a cell at time *t *= *τ*Δ*t*, we have

(7) $$s_{n}^{\tau +1}=\left(1-\Delta t\right)s_{n}^{\tau },$$

(8) $$\begin{array}{c} f_{n}^{\tau +1}=f_{n}^{\tau }+\left(B_{n}^{\tau }+\chi \left(\frac{1}{2}-s_{n}^{\tau }\right)f_{n}^{\tau }-{\left(f_{n}^{\tau }\right)}^{3}\right)\Delta t\\ +\sqrt{2\delta }\Delta W_{n}^{\tau } \end{array}$$

where the $\Delta W_{n}^{\tau }$ are independent random numbers drawn from a normal distribution with mean zero and variance Δ*t*.

The morphogen equations (3) are approximated numerically using a cell-centred finite-volume approach to discretise spatial derivatives. We denote by *a*_*j,k*_(*t*) and *b*_*j,k*_(*t*) (*j,k *= 1,..., *M*_*s*_) the average concentration of *a *or *b *in the region *I*_*j,k *_= [(*j*- 1)*h,jh*] × [(*k *- 1)*h, kh*] at time *t*, where *h *= *L/M*_*s*_. Equation (3a) becomes

(9) $$\begin{array}{l} \frac{d}{dt}(a_{j,k})=\frac{D_{a}}{h_{2}}(a_{j-1,k}+a_{j+1,k}+a_{j,k-1}+a_{j,k+1}\\ \;\;\;\;-4a_{j,k})+\frac{1}{h^{2}}\sum_{{\text{x}}_{n}\in I_{j,k}}\alpha_{a}(s_{n},f_{n})-\lambda_{a}a_{j,k} \end{array}$$

for 1 ≤ *j, k *≤ *M*_*s*_, and similarly for (3b).

Solutions to the continuous equations (3) have logarithmic singularities at the cell centres, as the cells are modelled as point sources. These singularities are regularised via the spatial discretization, which averages all quantities over a grid square, making the strength of autocrine signalling (and that between cells separated by distances which are of the order of *h *or less) dependent on *h*. The discrete equations are stepped forward in time using the Douglas alternating-direction implicit method [83,84]. The morphogen concentrations *a*(**x**_*n*_,t) and *b*(**x**_*n*_,t) experienced by the *n*-th cell are then taken to be those for the grid square in which its centre, **x**_*n*_, lies. As the system contains stochastic elements, we perform *M*_sim _simulation realisations for each set of parameter values.

The simulations were written in ISO C99, using the random number generator of the GSL library [85], and are available as Additional file 1.

### Spatial statistics

#### Pair correlation functions

PCFs are 'second-order' characteristics (involving relationships between pairs of points). We first define them for sets of points which are all of one type, before extending their definitions to the multitype case.

Let Π(***ξ***,***η***) be the probability of finding at least one cell centre in both of the infinitesimally small discs, with centres ***ξ ***and ***η ***and areas d*S*_1 _and dS_2_, respectively. The *product density *[41], *ρ*^(2) ^(***ξ***,***η***), is intuitively defined by Π(***ξ***, ***η***) = *ρ*^(2) ^(***ξ***,***η***) d*S*_1_d*S*_2 _(see [41,86] for a rigorous definition). If the pattern is translation-independent and isotropic, then *ρ*^(2) ^(***ξ***,***η***) ≡ *ρ*^(2) ^(*r*), where *r *= |***ξ ***- ***η***|. Let *ρ = N/L*^2 ^be the average density of cell centres. Then the PCF (or radial distribution function [87]) is defined by *g*(*r*) ≡ *ρ*^(2) ^(*r*)/*ρ*^2^, and describes the distribution of distances between pairs of cells.

In the multitype case, for each choice of *X, Y *∈ {*R*, G}, we define $\rho_{XY}^{\left(2\right)}\left(\xi ,\eta \right)$ as for *ρ*^(2) ^(***ξ***,***η***), except that we require the points in *S*_1 _and *S*_2 _to be of types *X *and *Y *respectively. The corresponding *cross pair correlation functions *[88] (or mark PCFs [41], or partial radial distribution functions [87]) are defined by $g_{XY}\left(r\right)=\rho_{XY}^{\left(2\right)}\left(r\right)∕\rho_{X}\rho_{Y}$, where *ρ*_*X *_is the density of cells of type *X*.

**Figure 9:**
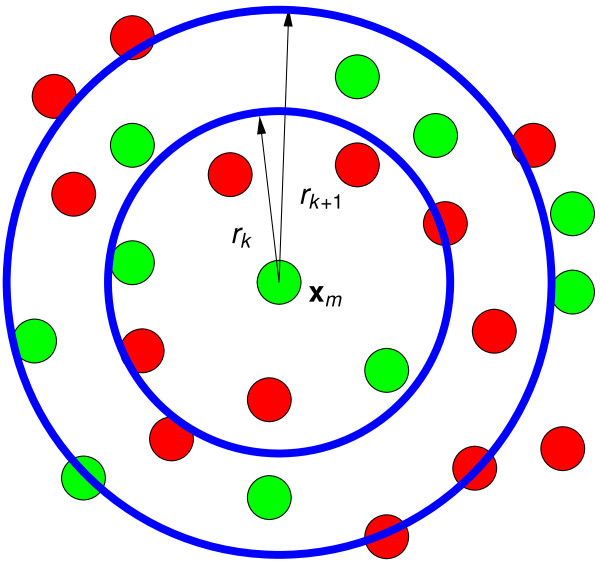
**Calculating PCFs**. Schematic diagram to illustrate the method used to calculate PCFs. For each distance interval (*r*_*k*_, *r*_*k*__+1_] and each cell with centre **x**_*m*_, we count the number of (other) cells in *r*_*k *_<*r *≤ *r*_*k*__+1 _where *r *is distance from **x**_*m*_. The PCF, *g*(*r*), on *r*_*k *_<*r *≤ *r*_*k+*__1 _is the mean number of cells in these annular regions normalised by $\pi \left(r_{k+1}^{2}-r_{k}^{2}\right)\rho$, which is the number of other cells which would be expected to be found in the annular region were the cells uniformly distributed (see equations (10)-(11)). For the cross PCFs *g_XY_*(*r*), we restrict **x**_*m *_to be of type *X *and only count cells of type *Y*; *g_S_*(*r*) is calculated from *g_RR_*(*r*) and *g_GG_(r) *by (12).

We estimate PCFs using the approach illustrated in Figure 9; see [41] (p. 284) for more detailed discussion. (Functions pcf for calculating *g*(*r*) and pcfcross for calculating *g_XY_*(*r*) are included in the R package spatstat [79].) A piecewise constant estimate of *g*(*r*) is obtained by dividing the range 0 <*r *<*L *into *M*_*g *_intervals of equal length *L/M*_*g*_. Setting *r*_*j *_*= jL/M*_*g*_, we approximate *g*(*r*) on *r*_*k *_<*r *≤ *r*_*k*__+1 _by

(10) $$g\left(r\right)=\frac{L^{2}}{N^{2}\pi \left(r_{k+1}^{2}-r_{k}^{2}\right)}\overset{N}{\underset{m=1}{\sum }}\overset{N}{\underset{n=1,n\neq m}{\sum }}I_{\left(r_{k},r_{k+1}\right]}\left(d_{nm}\right)$$

where *d*_*nm *_≡ | **x**_*n *_- **x**_*m *_|, *I*_(*s*,*t*]_(*r*) is the indicator function on (s,*t*]:

(11) $$I_{\left(s,t\right]}\left(r\right)=\left\{\begin{array}{cc} 1 & s<r\leq t,\\ 0 & \text{otherwise}. \end{array}\right)$$

For each cell *m *∈ {1, 2,..., *N*}, and each interval *k*, we calculate the number of cells in the annular region *r*_*k *_*< r *≤ *r*_k__*+*__1 _centred at **x**_*m*_, and normalise this by the expected number of cells in an area of this size were the cells to be uniformly distributed. We then average this over all *N *cells. (Smooth estimates of *g*(*r*) can be obtained by using a smoothing kernel in place of the indicator function.) Whilst the above estimate is piecewise constant, in order to show the distribution more clearly, we plot the values calculated as above at the centres of each interval ((*r*_k__*+*__1 _+ *r*_*k*_)/2) (this is linearly interpolated to give a continuous line).

The cross PCFs *g_XY _*are calculated in a similar manner, but the sums for *m *and *n *in (10) run only over cells of types *X *and *Y *respectively, and the normalization constant is $L^{2}∕\left[N_{X}N_{Y}\pi \left(r_{k+1}^{2}-r_{k}^{2}\right)\right]$, where *N*_*X *_and *N*_*Y *_are the numbers of cells of type *X *and *Y*. As the simulations are initially symmetrical in the two cell fates, we will combine *g_RR_*(*r*) and *g_GG_*(*r*) to give the cross PCF for pairs of cells of the same type, *g_S_*(*r*), defined by

(12) $$g_{S}\left(r\right)=\frac{{\left(\rho_{R}\right)}^{2}g_{RR}\left(r\right)+{\left(\rho_{G}\right)}^{2}g_{GG}\left(r\right)}{{\left(\rho_{R}\right)}^{2}+{\left(\rho_{G}\right)}^{2}}.$$

We choose to weight the two cross PCFs in proportion to the number of pairs of cells of that type, as *g_S_*(*r*)/*g*(*r*) is then the conditional probability that two randomly selected cells are of the same type, given that they are separated by a distance *r*, divided by the probability that any two randomly selected cells are of the same type $\left(\left(\rho_{R}^{2}+\rho_{G}^{2}\right)∕\rho^{2}\right)$. We take the arithmetic mean of PCFs over *M*_sim _realisations with the same parameter values in order to better estimate them.

#### Quadrat histograms

To calculate this statistic, we partition the domain [0, *L*] × [0, *L*] into *M*_*q *_× *M*_*q *_squares (or quadrats) with side length *L/M*_*q*_. We calculate the proportion *p*_*R *_of cells of type *R *(those for which *f*_*n *_> 0) in each quadrat, ignoring empty quadrats; we combine the results of *M*_sim _simulations with the same parameter values to generate a histogram of the distribution of *p*_*R *_over all quadrats and for all simulations.

## Competing interests

The authors declare that they have no competing interests.

## Authors' contributions

JAF developed the mathematical model in collaboration with HMB, OEJ and JRK. JAF also performed the numerical simulations and the statistical analyses of the resulting data. GRK generated the experimental results presented in Figure 1. All authors contributed to the preparation of the manuscript, and read and approved the final manuscript.

## Supplementary Material

Additional file 1 **Simulation source code**. Source code for simulations of pattern generation in populations of stem cells. Click here for file
